# Comparison of liver resection and transplantation for Child-pugh A cirrhotic patient with very early hepatocellular carcinoma and portal hypertension

**DOI:** 10.12669/pjms.305.5038

**Published:** 2014

**Authors:** Yan Dai, Chuan Li, Tian-Fu Wen, Lu-Nan Yan

**Affiliations:** 1 Yan Dai, Department of Day Surgery Center, West China Hospital of Sichuan University, Chengdu (610041), China.; 2 Chuan Li, Department of Liver Surgery & Liver Transplantation, West China Hospital of Sichuan University, Chengdu (610041), China.; 3 Tian-Fu Wen, Department of Liver Surgery & Liver Transplantation, West China Hospital of Sichuan University, Chengdu (610041), China.; 4 Lu-Nan Yan, Department of Liver Surgery & Liver Transplantation, West China Hospital of Sichuan University, Chengdu (610041), China.

**Keywords:** Hepatocellular carcinoma, Portal hypertension, Liver transplantation, Liver resection

## Abstract

***Objective: ***The aim of this study was to compare the outcomes of liver transplantation and resection for very early hepatocellular carcinoma (HCC, ≤ 2 cm) patients with Child-pugh A liver function and portal hypertension.

***Methods: ***From 2008 to 2013, 38 patients with Child-pugh class A liver function and portal hypertension were diagnosed as very early HCC, of whom 13 patients who received liver transplantation and 25 patients underwent liver resection. We compared the preoperative characteristics, recurrence-free survival (RFS) and overall survival (OS) rates of two group.

***Results:*** The baseline characteristics of two groups were similar. No perioperative mortality and liver failure were observed in both groups. The 1-, 3- and 5-year RFS rates of patients received liver resection and liver transplantation were 92.3%, 92.3% 92.3% and 92.0%, 71.7% and 64.5% respectively (*P*=0.140). The 1-, 3- and 5-year OS rates of two groups were also similar (100%, 91.7% and 91.7% for group T versus 100%, 93.3% and 93.3% for group R, *P*=0.695).

***Conclusion: ***Liver resection can offer satisfactory outcomes for very early HCC patients with well liver function and portal hypertension and should be considered as the first line choice for selected patients.

## INTRODUCTION

Hepatocellular carcinoma (HCC) is a leading cause of cancer related death in Asia, and its incidence is increasing in the Western countries.^1^^,^^2^ It is estimated that 8500 to 11,500 new cases of HCC occur annually in the United States, whereas the incidence of HCC in China has been reported to be more than 50 per 100,000.^3^^,^^4^

Liver resection and liver transplantation are two major curative treatments for HCC patients. Liver transplantation can cure not only HCC but also the background disease in the remnant liver. In 1996, Mazzaferro and colleagues proposed the Milan Criteria for liver transplantation for HCC.^5^ Patients within this criteria achieved 4-year recurrence-free and overall survival rates of 75% and 83% respectively.^5^ Subsequently, this excellent results were corroborated by many transplant centers.^6^^-^^8^ However, worldwide scarcity of deceased organ greatly limits widespread application of transplantation for patients with HCC. Approximately 20%-30% of candidates eventually dropped off the waiting list because of tumor progression.^1^^,^^9^ Compared with liver transplantation, liver resection removes only HCC; it cannot cure the underlying diseases. However, HCC frequently arises in the setting of cirrhosis which may cause portal hypertension. A number of investigations confirmed portal hypertension was associated with higher incidence of postoperative morbidity and poor long-term survival after liver resection.^10^^,^^11^ Some investigators even suggested portal hypertension is a contraindication for liver resection.^12^ Guidelines from both the American Association for the Study of Liver Diseases and the European Association for the Study of the Liver recommend liver transplantation for patients with portal hypertension, even when the liver function is in Child A class.^13^^,^^14^ However, according to the current allocation policy, model for end-stage liver disease (MELD) exception score is not allocated to patients with a HCC no more than 2 cm.^15^ So, patients with a HCC no more than 2 cm and normal liver function have no priority for liver transplantation.

Due to the high prevalence of hepatitis B virus infection, China alone accounts for approximately 55% of the HCC cases in the world.^16^ Only very few patients with HCC can receive liver transplantation for the extremely scarcity of organ in China, because of the scarcity of deceased organ. In the present study, we attempted to find out whether the outcomes of patients with a very early HCC (≤ 2 cm) and portal hypertension are acceptable after liver resection.

## METHODS

***Study group: ***Adult compensated cirrhotic (Child-Pugh A class) patients with portal hypertension and a single small HCC up to 2 cm in diameter who underwent liver transplantation or liver resection were recruited in this study. HCC was confirmed by pathology for all patients after operation. Patients were divided into two groups based on their treatment; group T consisted of patients who received liver transplantation, and group R consisted of patients who underwent liver resection. All transplantations and this study itself were approved by the ethical committee of West China Hospital.

***Data collection and follow-up: ***The following data were collected for each patient: age, gender, difference of treatments, tumor size, differentiation, microvascular invasion (MVI), total bilirubin (TB), albumin, platelets, alpha-fetoprotein (AFP), neutrophil-to-lymphocyte ratio (NLR). After operation, patients were regularly monitored by serum AFP examination, visceral ultrasonography or CT or MR imaging and chest radiography every three months. Bone scintigraphy was performed whenever HCC recurrence was suspected. Recurrence was defined as positive imaging findings compared to preoperative examination values and newly rising tumor marker (AFP) values or confirmation by biopsy or resection. All cases were regularly followed up to recurrence, death or the termination of this study. Significant portal hypertension (SPH) was defined as the presence of gastroeosophageal varices or splenomegaly (greater than 12 cm) with a platelet count of <100,000 cells/mm^3^.^17^ Postoperative ascites was defined as an abdominal drainage >500 mL/d or ascites needing medical treatment.^18^

***Statistical analysis: ***All continuous variables were presented as the mean ± SD and compared using one-way analysis of variance. Fisher’s exact test or the χ2 test was performed to compare categorical variables. Independent risk factors were identified by Cox regression. Factors significant at a* P*< 0.10 in the univariate analyses were included in the multivariate analyses. Recurrence-free and long-term survival rates were determined using the Kaplan-Meier method, with comparisons using the log-rank test. All analyses were performed using SPSS 21.0. We considered a *P* value of less than 0.05 to be significant.

## RESULTS

Among 38 patients with a single, small HCC and compensated liver function, 25 patients underwent liver resection (group R), whereas 13 patients received liver transplantation (group T, including 10 deceased donor liver transplantations and 3 living donor liver transplantations). This study included 34 males and 4 females. The mean age was 45.79±10.48 years. MVI was detected in 7 patients. Fifteen patients had a high preoperative AFP level. The mean follow-up of this study was 46.10±19.61 months. The mean tumor size was 1.74±0.46 cm for group T and 1.80±0.28 cm for group R. Five patients in group R received synchronic liver resection and splenectomy because of moderate or severe hypersplenism (defined as white blood cell counts of less than 2.0×10^9^/L and/or platelet count below 50×10^9^/L and/or splenomegaly classified as grade II or higher^17^). As presented in Table-I, there was no difference in sex distribution, preoperative total bilirubin (TB), albumin, platelet levels, differentiation, presence of MVI between two groups. No in-hospital mortality was observed in both groups. In group R, three patients suffered from ascites after resection. These three patients recovered after using diuretic. No liver failure was observed in group R.

**Fig.1:**
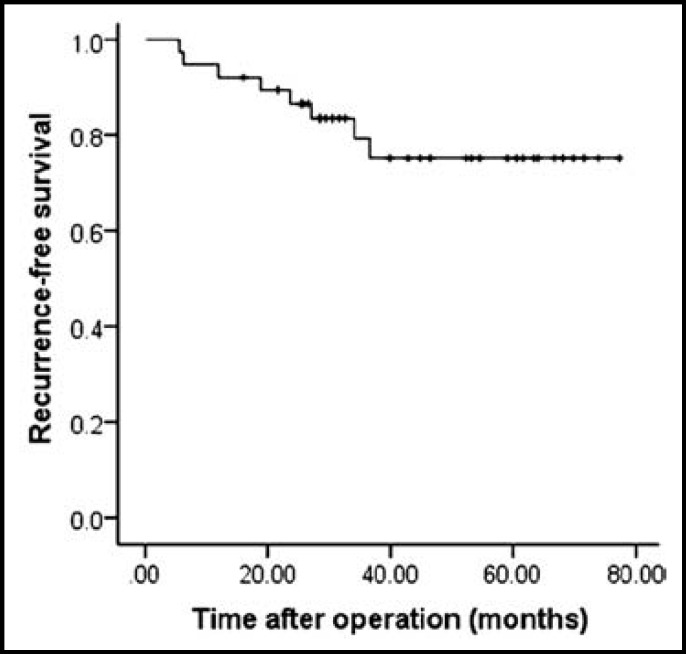
The recurrence-free survival curves for all of the patients

**Fig.2:**
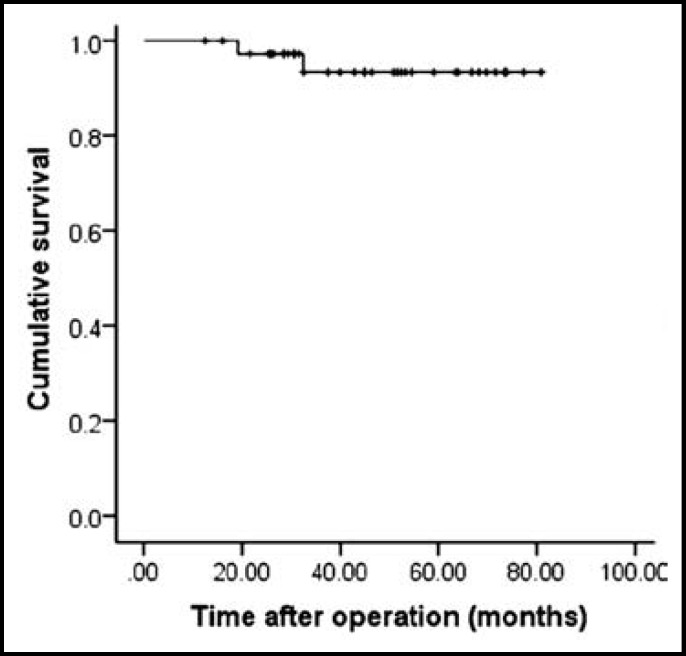
The long-term survival curves for all of the patients

***Recurrence and survival outcomes of the two groups: ***Postoperative recurrence was observed in 8 patients, including 1 patient in group T and 7 patients in group R. Of the 8 patients with recurrence, 3 patients received radiofrequency ablation, and 5 patients received transcatheter arterial chemoembolisation. During the follow-up period, one patient in the group R and one patient in the group T died of recurrence. The 1-, 3-, 5-year RFS rates for the entire cohort were 92.1%, 79.3% and 75.2% (Fig.1), whereas the 1-, 3- and 5-year OS rates of all patients were 100%, 93.3% and 93.3% (Fig.2).

**Fig.3:**
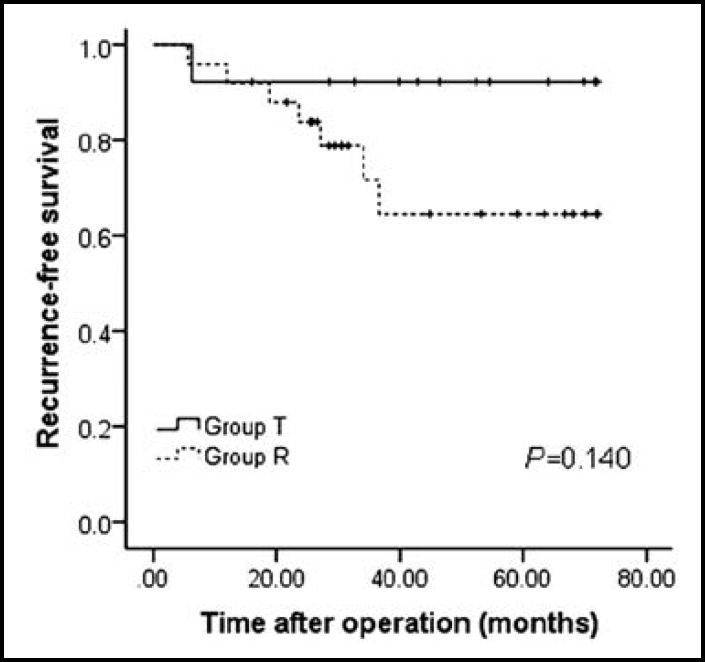
Recurrence-free survival for Child-pugh class A patients with very early HCC and portal hypertension undergoing liver transplantation versus liver resection

**Fig.4:**
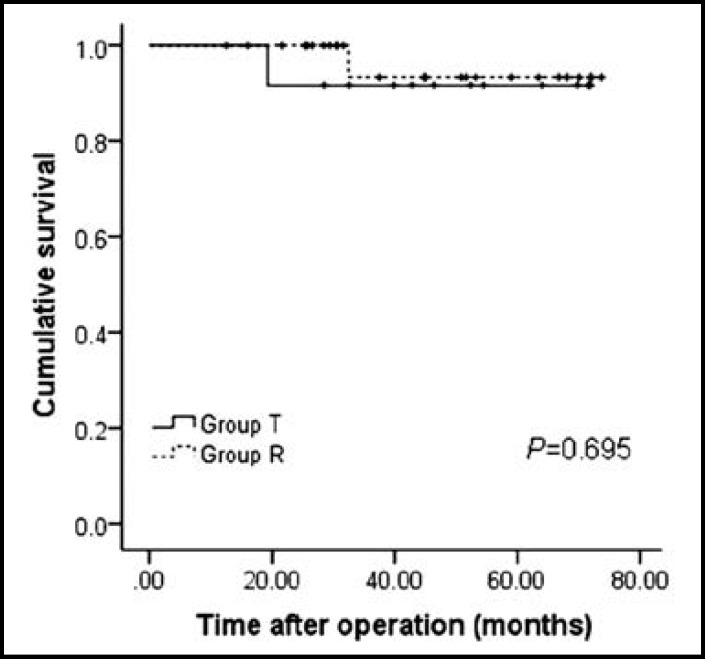
Long-term survival for Child-pugh class A patients with very early HCC and portal hypertension undergoing liver transplantation versus liver resection

We compared the recurrence-free and overall survival rates of patients who underwent liver transplantation versus resection. The 1-, 3- and 5-year RFS rates of patients received liver resection were lower than patients underwent liver transplantation (92.3%, 92.3% and 92.3% for group T versus 92.0%, 71.7% and 64.5% for group R, Fig.3; *P*=0.140). However, this difference didn’t reach statistical significance. Moreover, the 1-, 3- and 5-year OS rates of two groups were similar (100%, 91.7% and 91.7% for group T versus 100%, 93.3% and 93.3% for group R, Fig.4; *P*=0.695).

## DISCUSSION

The management for patient with small HCC and portal hypertension is under debate. Although patient with small HCC and portal hypertension after liver transplantation have an excellent outcomes, there is a great imbalance of graft available and need.^19^ In our study, we confirmed patient with a very early HCC and portal hypertension after liver resection could achieve a satisfactory long-term survival which is similar to liver transplantation. According to the current allocation policy, patient with a ≤ 2 cm HCC no longer receive any model for end-stage live disease exception points.^15^ Therefore, patient with very early HCC and preserve liver function don’t have any advantage to be allocated a graft according to current allocation policy. Our study suggested for such patient, liver resection should be considered as the first choice for the treatment for very early HCCs, even accompany with portal hypertension.

**Table-I:** Baseline demographic characteristics in the two groups

***Variables ***	***Group T (N=13)***	***Group R (N=25)***	***P***
Age (year)	45.54±11.26	45.61±20.05	0.750
Female/male	1/12	3/22	0.681
Tumor size (cm)	1.74±0.46	1.80±0.28	0.104
MVI	4	3	0.203
Differentiation			0.537
Well	3	3	
Moderate	8	15	
Poor	2	7	
AFP ＞ 400ng/mL	4	11	0.501
TB (μmol/L)	16.88±7.71	16.51±5.51	0.339
Albumin (g/L)	42.65±4.55	41.43±3.57	0.215
Platelet (10^9^/L)	65.85±22.99	71.12±18.31	0.385

The 5-year survival rate of patients with a very early HCC and portal hypertension after liver resection was 93.3% in the current study. However, in Roayaie et al’s study, the 5-year survival rate of patients with a ≤ 2 cm HCC after resection was about 70%.^20^ The recurrence rate of group R in our study is also lower than Roayaie et al’s study.^20^ The proportion of patient with MVI in our study is 12% (3/25) which is much lower than Roayaie et al’s study (27%).^20^ A number of studies have confirmed the overall survival and recurrence-free survival for very early HCC were significantly affected if MVI was detected by pathological examination. Moreover, the background hepatic disease of all patients in our study is hepatitis B virus infection, whereas the main cause of liver cirrhosis in their study is hepatitis C infection.^20^ Kao et al.^21^ also suggested the long-term survival rate was better in patients with hepatitis B related HCC than patients with hepatitis C associated HCC in the cases of transplantable and Barcelona-Clinic Liver Cancer stage A HCC. These differences may explain why our outcomes were better than their study.

No perioperative mortality was observed in this study. However, a number of studies have suggested that clinically significant portal hypertension was associated with increased incidence of postoperative complications and poor outcomes after liver resection.^10^^,^^12^ Current guidelines from both the American Association for the Study of Liver Diseases and the European Association for the Study of the Liver don’t recommend that liver resection for patients with portal hypertension.^13^^,^^14^ Our results did not support this recommendation and confirmed that liver resection for patients with portal hypertension and very early HCC can achieve a long-term survival rate similar to that of liver transplantation. We would like to emphasise that the guidelines of the two above-mentioned associations were based on very early studies.^12^^,^^22^ Technologies for liver resection and perioperative management have improved. In our clinical practice, we emphasise the assessment of liver volume before liver resection for patients with portal hypertension. We do not perform liver resection for patients with significant reduction in liver volume. Recently, Giannini et al.^23^ confirmed clinically significant portal hypertension had no negative impact on the outcomes of patients with well liver function undergoing liver resection for patients with a HCC up to 5 cm. Santambrogio et al.^24^ also suggested clinically significant portal hypertension is not a contraindication for cirrhotic patients with HCC. Moreover, for patients with severe hypersplenism, we suggest synchronic liver resection and splenectomy. Chen et al.^25^ suggested that simultaneous liver resection and splenectomy could improve the long-term recurrence-free survival of patients with HCC and hypersplenism.

There were some limitations in our study. The sample size of our study is small. There were only 13 patients in the transplantation group and 25 patients in the resection group. In our study, the definition of portal hypertension was based on clinical findings without the measurement of hepatic venous pressure gradient. Assessment of hepatic venous pressure gradient before operation is an invasive management. For the culture reason, this management cannot be conventional used in our country. This was a retrospective study which was carried out by one single center. However, for the ethical consideration, randomized-controlled study cannot be performed. We suggested a multicenter study design would minimize these limitations.

In conclusion, there was no marked difference in the overall survival and recurrence-free survival rates between liver transplantation and resection for patients with very early HCC and portal hypertension. Liver resection should be considered as the first line choice for patients with very early HCC and portal hypertension.
